# 

**Published:** 1975-01

**Authors:** 


					\Jj t
?/?3J3
BRISTOL
MEDICO-
CHIRURGICAL
JOURNAL
?
JANUARY 1975
3 33
?ji ?<;? 3 y^VXoXald
VOL. 90 (i) No.< 334

				

